# Surrogate indices of insulin resistance using the Matsuda index as reference in adult men—a computational approach

**DOI:** 10.3389/fendo.2024.1343641

**Published:** 2024-04-23

**Authors:** Víctor Antonio Malagón-Soriano, Andres Julian Ledezma-Forero, Cristian Felipe Espinel-Pachon, Álvaro Javier Burgos-Cárdenas, Maria Fernanda Garces, Gustavo Eduardo Ortega-Ramírez, Roberto Franco-Vega, Jhon Jairo Peralta-Franco, Luis Miguel Maldonado-Acosta, Jorge Andres Rubio-Romero, Manuel Esteban Mercado-Pedroza, Sofia Alexandra Caminos-Cepeda, Ezequiel Lacunza, Carlos Armando Rivera-Moreno, Aquiles Enrique Darghan-Contreras, Ariel Iván Ruiz-Parra, Jorge E. Caminos

**Affiliations:** ^1^ Department of Internal Medicine, School of Medicine Universidad Nacional de Colombia, Bogotá, Colombia; ^2^ Department of Physiology, School of Medicine Universidad Nacional de Colombia, Bogotá, Colombia; ^3^ Endocrinology Unit - Department of Internal Medicine, School of Medicine Universidad Nacional de Colombia, Bogotá, Colombia; ^4^ Department of Obstetrics and Gynecology, School of Medicine Universidad Nacional de Colombia, Bogotá, Colombia; ^5^ School of Medicine, Universidad Pompeu Fabra, Barcelona, Spain; ^6^ Centro de Investigaciones Inmunológicas Básicas y Aplicadas (CINIBA), Facultad de Ciencias Médicas, Universidad Nacional de La Plata, La Plata, Argentina

**Keywords:** surrogate indices, insulin resistance, young adult men, computational approach, Matsuda index

## Abstract

**Background:**

Overweight and obesity, high blood pressure, hyperglycemia, hyperlipidemia, and insulin resistance (IR) are strongly associated with non-communicable diseases (NCDs), including type 2 diabetes, cardiovascular disease, stroke, and cancer. Different surrogate indices of IR are derived and validated with the euglycemic–hyperinsulinemic clamp (EHC) test. Thus, using a computational approach to predict IR with Matsuda index as reference, this study aimed to determine the optimal cutoff value and diagnosis accuracy for surrogate indices in non-diabetic young adult men.

**Methods:**

A cross-sectional descriptive study was carried out with 93 young men (ages 18–31). Serum levels of glucose and insulin were analyzed in the fasting state and during an oral glucose tolerance test (OGTT). Additionally, clinical, biochemical, hormonal, and anthropometric characteristics and body composition (DEXA) were determined. The computational approach to evaluate the IR diagnostic accuracy and cutoff value using difference parameters was examined, as well as other statistical tools to make the output robust.

**Results:**

The highest sensitivity and specificity at the optimal cutoff value, respectively, were established for the Homeostasis model assessment of insulin resistance index (HOMA-IR) (0.91; 0.98; 3.40), the Quantitative insulin sensitivity check index (QUICKI) (0.98; 0.96; 0.33), the triglyceride-glucose (TyG)-waist circumference index (TyG-WC) (1.00; 1.00; 427.77), the TyG-body mass index (TyG-BMI) (1.00; 1.00; 132.44), TyG-waist-to-height ratio (TyG-WHtR) (0.98; 1.00; 2.48), waist-to-height ratio (WHtR) (1.00; 1.00; 0.53), waist circumference (WC) (1.00; 1.00; 92.63), body mass index (BMI) (1.00; 1.00; 28.69), total body fat percentage (TFM) (%) (1.00; 1.00; 31.07), android fat (AF) (%) (1.00; 0.98; 40.33), lipid accumulation product (LAP) (0.84; 1.00; 45.49), leptin (0.91; 1.00; 16.08), leptin/adiponectin ratio (LAR) (0.84; 1.00; 1.17), and fasting insulin (0.91; 0.98; 16.01).

**Conclusions:**

The computational approach was used to determine the diagnosis accuracy and the optimal cutoff value for IR to be used in preventive healthcare.

## Introduction

The world population with obesity [body mass index (BMI) ≥ 30 kg/m^2^] in the year 2020 was 988 million individuals (14%), and by 2035, it is projected to reach 1.914 billion people (24%) (1). Moreover, according to the statistics of the International Diabetes Federation (IDF), approximately 537 million adults between the ages of 20 and 79 worldwide have diabetes, and it is expected to reach 643 million by the year 2030 (2). Additionally, obesity is associated with chronic and low-grade inflammation due to the abnormal or excessive fat secretions of adipokines that might lead to decreases in insulin sensitivity in target tissues, such as adipose tissue, skeletal muscle, and liver (3, 4). The insulin resistance (IR) or impaired insulin sensitivity is considered to be one of the major invisible changes, between 10 and 15 years, before the diagnosis and progression of different non-communicable diseases (NCDs), including type 2 diabetes (T2DM), nonalcoholic fatty liver disease (NAFLD), heart disease, and stroke (3–5).

On the other hand, progression to hyperglycemia and T2DM may be caused by impaired insulin secretion due to beta cell dysfunction or insulin insensitivity of target tissues (6, 7). In patients with diabetes mellitus, chronic hyperglycemia and IR are risk factors for the pathogenesis of atherosclerosis and long-term cardiovascular complications and, therefore, the main cause of disability and death. It is important to highlight that the diagnosis of IR and T2DM is based on determinations of fasting glucose and glycated hemoglobin (HbA1c) levels and 2-h post-load plasma glucose (2h-PG) measurements after an oral glucose tolerance test (OGTT), methodology that in many circumstances cannot detect this pathology in the early stages, as described elsewhere (8). On the other hand, the Matsuda index, the Homeostasis model assessment of insulin resistance index (HOMA-IR), and the Quantitative insulin sensitivity check index (QUICKI) are the most common surrogate indices with the highest accuracy for evaluating insulin sensitivity/resistance and showed a strong significant correlation with the clamp-derived insulin sensitivity [euglycemic–hyperinsulinemic clamp (EHC)] test (6, 9). Additionally, previously studies have demonstrated that the Matsuda index determined after an OGTT, which combines both hepatic and peripheral tissue insulin sensitivity analysis, has greater diagnostic ability than the HOMA-IR index, which is based on fasting analysis samples and is associated primarily with hepatic IR (9, 10). Moreover, fasting glucose, 1h-PG, and 2h-PG have been studied to predict IR and their relation with several parameters used in diagnosing IR in diverse NCDs (8, 11). In this way, there is an urgent need to establish the most predictive IR index with excellent sensitivity, specificity, and optimal cut-off value for health impact assessment in chronic NCDs.

On the other hand, several surrogate indices have been proposed in population studies that are based on anthropometric, biochemical, and hormonal determinations to assess insulin sensitivity/resistance using reliable, accessible, and less expensive methods. However, owing to the high cutoff value variability observed, additional studies are required to validate the reliable cutoff values of these indices for detecting IR (12, 13). Additionally, previous studies have shown that the use of fasting and 2h-PG levels has relatively low accuracy for early prediction of impaired glucose tolerance and T2DM, cardiovascular disease (CVD), and mortality rate (14). In this way, several surrogate indices that evaluate IR have been validated, including Matsuda, HOMA-IR, QUICKI, triglyceride-glucose (TyG) index, triglycerides-to-HDL-C ratio (TG/HDL-c), BMI (kg/m^2^), visceral adiposity index (VAI), TyG-waist circumference (TyG-WC), TyG-body mass index (TyG-BMI), TyG-waist-to-height ratio (TyG-WHtR), lipid accumulation product (LAP), leptin/adiponectin ratio (LAR), total body fat percentage (TFM %), android fat (AF %), waist circumference (WC), and waist-to-height ratio (WHtR) (12–23).

Obesity-associated IR as a risk factor that may increase the progression to prediabetes, T2DM, and CVD as the leading cause of global death, and prompt implementation of accurately surrogate indices might be used as predictive tools for population-based screening programs for recommendations of preventive action to address and mitigate NCDs and to reduce the period of undiagnosed diabetes and complications at the time of diagnosis, several years before the onset of symptoms. The EHC technique is the gold standard method for the detection of IR with limited clinical applicability; however, different surrogate indices of IR have been proposed, and some of these values remain dubious due to the lack of standard, desirable, and local cutoff value guidelines for early detection to improve the diagnosis and treatment of disorders associated with hyperglycemia and IR (9, 24).

Thus, the aims of this study are to propose a computational approach to accurately determine the optimal cutoff values and the ability to predict IR using the Matsuda index as reference for surrogate indices of insulin sensitivity/resistance in non-diabetic young adult men, and to use this approach quickly, easily, and at a low cost for IR screening and preventive medicine.

## Materials and methods

### Ethical considerations

This protocol was approved by the Ethics Committee of the School of Medicine—Universidad Nacional de Colombia (protocols B.FM.1.002-CE-0194-22 and B.FM.1.002-CE-081-22) and conducted in accordance with the Helsinki Declaration. All individuals were informed about the aim of this research study and gave their written consent prior to enrollment in the protocol study. The inclusion criteria were as follows: lean (BMI between 18.0 and 24.9 kg/m^2^) and obese (BMI ≥ 30 kg/m^2^) young adult men (18–31 years of age). Participants with preexisting metabolic diseases, T2DM, liver disease, and renal and cardiovascular dysfunction who were taking thyroid medications and current therapy that could alter metabolism were excluded.

### Study design and participants

The methodology of the current study has been described in detail elsewhere (25). Briefly, an exploratory cross-sectional study with case and control selection of the individuals (obese and healthy men) was conducted with 93 young adult men (ages 18–30). Weight, height, WC, systolic blood pressure (SBP, mmHg), and diastolic blood pressure (DBP, mmHg) were determined by trained personnel. Body composition, including TFM (%), gynoid fat (GF, %), and central fat mass (AF, %), was obtained by dual energy x-ray absorptiometry (DXA) (Lunar Prodigy Primo - GE Healthcare). BMI was determined as weight (kg) divided by height (m) squared (kg/m^2^).

### Analytical assessment

All subjects underwent a 75-g OGTT after an overnight fast of 8–10 h. Blood samples were drawn in a dry tube from an antecubital vein between 7:00 and 8:00 a.m. during fasting before glucose ingestion (0 h) and 30, 60, and 120 min after a 75-g oral glucose load. Samples were centrifuged (4,000*g*) and serum was transferred into plastic tubes and stored at −80°C until analysis. At each point, glucose and insulin were determined; meanwhile, lipid profile and leptin and adiponectin levels were analyzed in fasting state, as described elsewhere (25). Leptin and adiponectin levels were determined by ELISA, as described previously (25).

### Calculation of indices

The Matsuda index, HOMA-IR, QUICKI, TyG index, TG/HDL-c, VAI, TyG-WC, BMI (kg/m^2^), TyG-BMI, WHtR, TyG-WHtR, LAP, and LAR were calculated as described elsewhere (12, 22, 26–33). Formulas for surrogate indices are described in the **Supplementary Materials**.

### Statistical analysis

The average ± standard deviation (SD) for each variable was presented in a tabular array. Initially, a descriptive analysis was performed separating groups into IR and non-IR levels, with the Matsuda index as reference. IR was defined according to the cutoff value of the Matsuda index as described elsewhere (13, 15). The optimal starting cutoff value for Matsuda was generated from the arithmetic average of the maximum of the IR level with the minimum of the non-IR level (in this case, the optimal starting value was 4.03).

For the bivariate analysis, a scatter diagram was made to detect the monotonic or linear pattern of the relationship of the variables and to describe the windows of separation or overlap of importance to define the starting point of the algorithm or initial conditions. Thus, bivariate analyses for the Matsuda index and glucose, insulin levels, hormonal levels, anthropometric measures, and different surrogate indices were also performed. Furthermore, the violin plots showing the interquartile range distribution of IR and non-IR individuals based on the optimal cutoff value and diagnosis performance (sensitivity and specificity) were obtained using the iterative computational approach process for the different variables. The R code for both the computational proposal and the final statistical analysis appears on the GitHub described in the **Supplementary Materials**.

### Computational approach description

The computational approach for diagnosis accuracy and determination of the optimal cutoff values for prediction of insulin sensitivity/resistance is described in the **Supplementary Materials**. The key steps involved in the algorithm approach are listed below; however, in the **Supplementary Materials**, the code is developed and commented on so that any user can understand each step until the output is generated:

**1.** Standardize by *Z*-score (SD) all quantitative variables: This was done because of the large differences in the variances of each variable (34).

**2.** Generation of the midpoints of the window of separation (Matsuda) and overlap or separation for the second variable in the bivariate scatter plot: Since Matsuda was an index that clearly separated the groups related to IR perfectly, the separation window was called the situation that occurred with this index, where the window corresponded to the maximum in the IR group and the minimum in the non-IR group. The overlap window occurs when the maximum of the IR group falls above the minimum of the non-IR group; in this case, the midpoint of these extremes was also constructed but the window was called an overlap window.

**3.** A matrix of spatial weights. This was done to give a spatial connotation to the observations, looking for those closest to the separation or overlapping windows to have the highest weight, since, at the intersection of these two regions, there is an area with the highest possibility of misclassifying an observation. The staging in this case was min–max [0,1] (unity-based normalization) so that the weights would fall in this range. In addition, once standardized, the weights matrix was standardized by rows so that the sum of each row would yield a total weight equal to unity (35, 36).

**4.** Spearman’s correlation coefficient (*r*). The scatter diagram between the Matsuda index and any other with which it was contrasted evidenced a monotonic relationship that is not necessarily linear. In this sense, this correlation coefficient was used as a weighting in the components of the objective function, because from a spatial context, the coordinate in the abscissa or the ordinate may have different importance, so the quadratic spearman correlation coefficient acts as a weighting, since the higher the value of this measure, the greater the weight given to this coordinate.

**5.** Generation of the new coordinates of the initial cutoff point. With the weights and midpoints of separation or overlap windows, the vector of coordinates is generated in “*x*” (for Matsuda) and coordinates are generated in “*y*” (for the index with which the bivariate dispersion diagram is made). The new coordinates are given by the vectors:


$$\;x_{o}=\;r^{2}WX_{z}+\left(1-r^{2}\right)X_{z}$$

$$\;y_{o}=\;r^{2}WX_{z}+\left(1-r^{2}\right)Y_{z}$$

where W represents the weight matrix, r represents the Spearman correlation coefficient, $X_{z}$ and $Y_{z}$ are the original standardized variables corresponding respectively to the Matsuda index and the other variable with which the scatter diagram is made.

**6.** Then, the distance ( d ) between the average of the coordinates of $\;x_{o}$ and $y_{o}$ is calculated with the midpoint of the separation window (Matsuda) and the midpoint of the separation window or overlap of the variable with which bivariate dispersion is generated. The expression for this distance is given by:


$$d=\sqrt{{\left(\bar{x_{o}}-x_{w}\right)}^{2}+{\left(\bar{y_{o}}-y_{w}\right)}^{2}}$$

where $x_{w}$ and $y_{w}$ represent the midpoints of the separation window and the overlap or separation window (if applicable), respectively. As the construction of the weight matrix W was initially defined as the inverse of the distances between all points standardized in min–max mode, in the literature, the possibility of raising the weight matrix to a power *p* appears (in this case in values from 0.50 to 2.50 in step of 0.01) so that it can be verified if with these new matrices of iterative weights a distance less than that established with *p* = 1 can be obtained (the usual case and the one represented in the equations described above).

**7.** To give greater robustness to the algorithm, an iteration block is proposed where it is removed (with replacement) one by one from the observations. This process is repeated *n* times, where *n* is the number of rows in the data matrix.

The pseudocode for this iterative procedure could be:

Pseudocode:

**Table:** 

for *j* in range(start = 1, end = 93, step = 1): datos = datos(without *j* row) W = “preprocessing” for *p* in range(start = 0.5, end = 2.5, step = 0.01) x, y = cutoff by *p* end for xm, ym = cutoff with minimum distance return Youden index end for cutoff selected = maximum Youden index

**8.** For the minimum distance of the first iterative process and the maximum Youden of the second process after the calculations of the confusion matrix, with sensitivity and specificity with the cutoff point obtained from the minimum distance, the optimal cutoff point is obtained since the Jackknife process (first order) could have adjusted the data coordinate vectors of the two variables that make up the scatter plot.

**9.** Finally, for each variable contrasted with Matsuda, the cutoff points as well as the sensitivity and specificity values are recorded.

## Results

**Figure 1:**
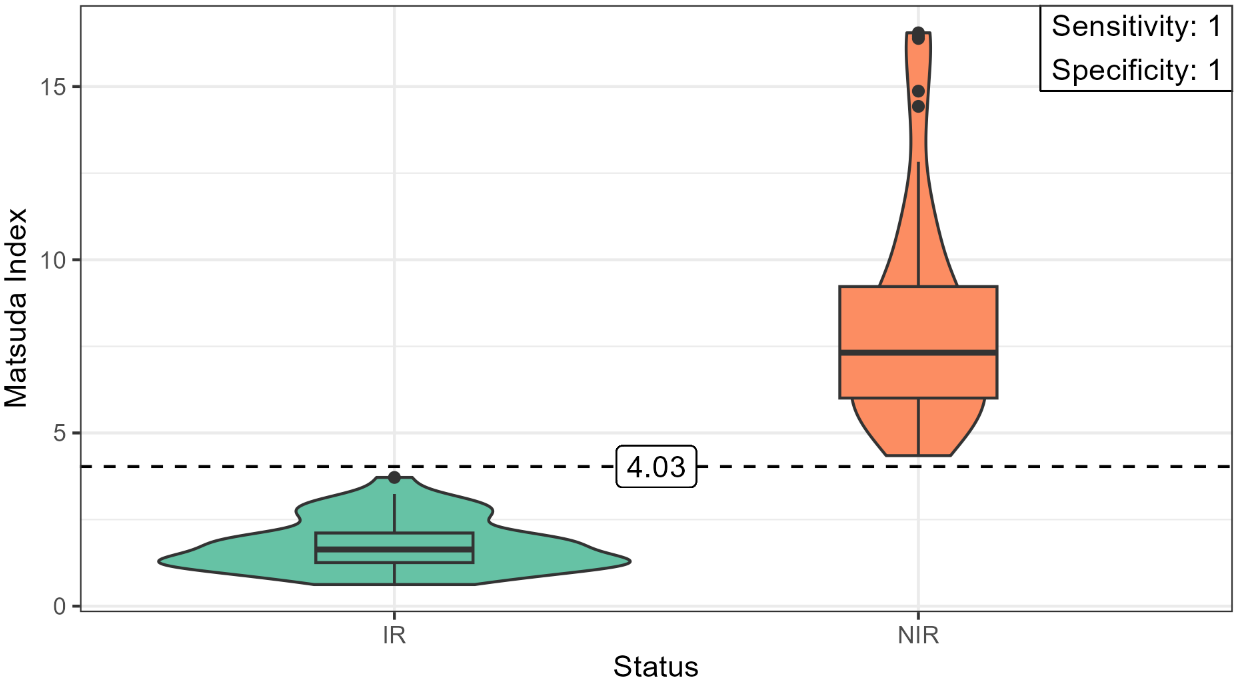
The figure shows the violin plot of the distribution of IR and non-IR individuals in relation to the cutoff value (4.03) and the diagnosis performance (sensitivity and specificity) for the prediction of IR determined using the algorithmic approach for the Matsuda index. Low values of the Matsuda index are associated with the risk of IR. The violin plots show the interquartile range distribution of IR and non-IR individuals.

The characteristics of the study participants are described in **Table 1**. Considering the cutoff values for the Matsuda index, the individuals were initially classified into insulin resistant (IR) and non-IR, as described elsewhere (13, 15). Then, the optimal cutoff value for the Matsuda index (4.03) was obtained employing progressive iterative approximation using the computational approach until it reaches the cutoff value threshold (**Figure 1**).

**Table 1:** Characteristics of non-insulin and insulin-resistant (IR) individuals.

Variable	Non-insulin-resistant individuals* (*n* = 48)	Insulin-resistant (IR) individuals* (*n* = 45)
**Age (years), mean (range)**	23 (18–30)	24 (18–31)
**Body mass index (BMI) (kg/m^2^)**	21.6 ± 1.9 (17.6–25.8)	36.3 ± 4.8 (30.5–48.3)
**Height (cm)**	172.5 ± 5.8 (161–184)	173.8 ± 6.8 (155–192)
**Waist circumference (WC) (cm)**	76.2 ± 5.2 (67–87)	108.7 ± 8.1 (96–128)
**Hip circumference (HC) (cm)**	93.0 ± 5.8 (78.2–104.1)	119.6 ± 10.1 (100.0–146.0)
**Waist-to-height ratio (WHtR)**	0.4 ± 0.0 (0.4–0.5)	0.6 ± 0.1 (0.5–0.8)
**Total fat mass** **(TFM %)**	19.0 ± 5.8 (7.1–30.1)	42.5 ± 4.8 (32.7–53.8)
**Android fat (AF%)**	25.2 ± 8.3 (10.5–44.1)	53.5 ± 4.3 (43.5–62.4)
**Gynoid fat (GF %)**	25.0 ± 5.5 (11.9–34.8)	45.0 ± 5.1 (35.3–55.8)
**[AF (%)/GF (%)] ratio**	1.0 ± 0.2 (0.6–1.3)	1.2 ± 0.1 (1.1–1.4)
**Systolic blood pressure (SBP) (mmHg)**	111.0 ± 12.4 (90.0–148.0)	129.0 ± 12.5 (110.0–152.0)
**Diastolic blood pressure (DBP) (mmHg)**	70.3 ± 8.8 (50.0–90.0)	84.0 ± 10.6 (60.0–102.0)
**Mean blood pressure (MBP) (mmHg)**	83.9 ± 8.6 (67.0–108.0)	98.9 ± 10.4 (80.0–119.0)
**Fasting glucose (mg/dL)**	82.7 ± 7.3 (69–98)	89.5 ± 11.3 (74–122)
**Glucose (mg/dL) 30′ OGTT**	109.4 ± 19.4 (71.0–156.0)	135.5 ± 24.2 (95.0–201.0)
**Glucose (mg/dL) 60′ OGTT**	81.8 ± 16.0 (53–115)	116.1 ± 31.3 (56–201)
**Glucose (mg/dL) 120′ OGTT**	74.0 ± 12.0 (53–101)	91.2 ± 24.8 (50–149)
**Fasting insulin (µIU/mL)**	6.4 ± 2.9 (2.5–17.3)	27.4 ± 10.9 (12.5–58.1)
**Insulin 30′ OGTT (µIU/mL)**	63.5 ± 37.6 (2.5–192.5)	233.3 ± 107.8 (46.3–497.5)
**Insulin 60′ OGTT (µIU/mL)**	39.5 ± 20.5 (9.5–92.0)	170.4 ± 91.4 (35.3–406.5)
**Insulin 120′ OGTT (µIU/mL)**	23.7 ± 12.0 (4.8–62.5)	99.8 ± 81.3 (10.7–377.9)
**Triglycerides (mg/dL)**	90.1 ± 30.7 (47.0–174.0)	177.1 ± 79.4 (55.0–398.0)
**Total cholesterol (mg/dL)**	161.0 ± 33.0 (93.0–254.0)	186.1 ± 26.8 (127.0–245.0)
**HDL- cholesterol (mg/dL)**	48.4 ± 7.2 (34.0–65.0)	42.4 ± 9.6 (31.0–76.0)
**Leptin (ng/mL)**	7.6 ± 0.7 (6.5–10.1)	27.2 ± 13.1 (14.4–78.4)
**Adiponectin (µg/mL)**	15.3 ± 1.8 (11.5–19.1)	13.3 ± 1.9 (8.9–17.6)
**LAR (ng/µg)**	0.5 ± 0.7 (0.4–0.7)	2.0 ± 0.9 (1.0–5.6)
**Matsuda**	8.3 ± 3.2 (4.3–16.6)	1.8 ± 0.8 (0.6–3.7)
**HOMA-IR**	1.3 ± 0.6 (0.5–3.4)	6.1 ± 2.8 (2.3–13.9)
**QUICKI**	0.4 ± 0.0 (0.3–0.4)	0.3 ± 0.0 (0.2–0.3)
**TyG**	4.4 ± 0.2 (4.1–4.9)	4.8 ± 0.3 (4.2–5.3)
**TG/HDL-c**	1.9 ± 0.7 (1.0–4.1)	4.2 ± 1.9 (0.7–10.3)
**TyG-WC**	339.6 ± 30.5 (274.5–403.7)	518.1 ± 49.1 (433.5–632.1)
**TyG-WHtR**	1.8 ± 0.2 (1.6–2.4)	3.0 ± 0.3 (2.4–3.7)
**TyG-BMI**	95.8 ± 10.8 (71.6–121.9)	171.8 ± 23.5 (141.2–238.9)
**LAP**	12.4 ± 8.0 (1.4–31.4)	86.5 ± 42.5 (23.6–201.9)
**VAI**	2.3 ± 0.9 (0.9–5.2)	5.3 ± 2.6 (0.9–12.8)

*Insulin sensitivity/resistance was determined using the Matsuda index cutoff value (4.03) as reference. Homeostatic model assessment index (HOMA-IR), Quantitative insulin sensitivity check index (QUICKI), triglyceride-glucose (TyG) index, triglycerides-to-HDL-c ratio (TG/HDL-C), visceral adiposity index (VAI), TyG-waist circumference (TyG-WC), TyG-body mass index (TyG-BMI), TyG-waist-to-height ratio (TyG-WHtR), lipid accumulation product (LAP), leptin/adiponectin ratio (LAR), total fat mass (TFM %), android fat (AF %), body mass index (BMI) (kg/m^2^), waist circumference (WC), and waist-to-height ratio (WHtR). Data are mean ± SD and range in parentheses.

It is important to highlight that QUICKI, BMI (kg/m^2^), total body fat (TBF %), AF (%), WC (cm), TyG-WHtR, WHtR, leptin levels, and LAR have been described as predictors of insulin sensitivity/resistance in young male adults, yielding similar results to those described in **Table 1** in the individuals grouped into IR and non-IR in this study, findings that confirm the high diagnosis accuracy classification using the Matsuda index cutoff value as reference (4.03) when the computational approach is applied for IR discrimination (**Table 1**) (21, 22, 37–44). Additionally, anthropometric measurement, clinical features, leptin, lipid profile, glucose, and insulin levels during fasting at each point of the OGTT and surrogate indices of muscle and hepatic insulin sensitivity are described in IR and non-IR young individuals as described in **Table 1**.

**Figure 2:**
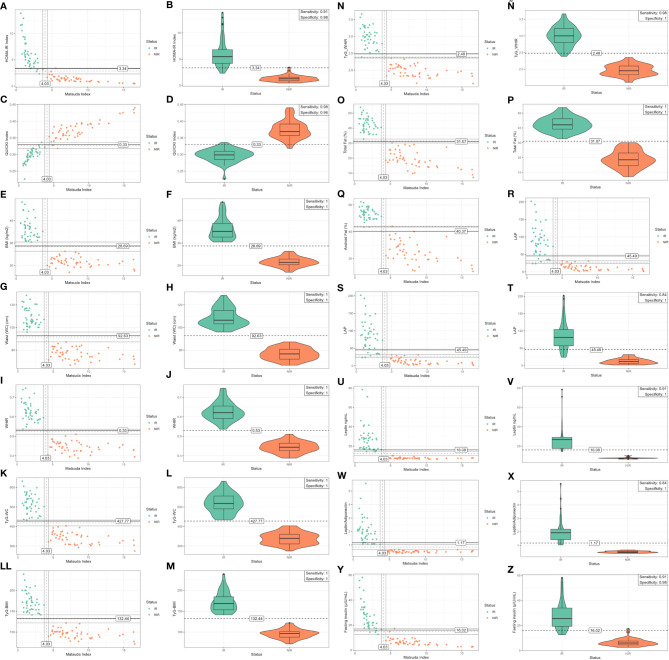
The figures in the left column **(A, C, E, G, I, K, LL, N, O, Q, S, U, W, Y)** show the scatter plot depicting the bivariate distribution segregated by the status of IR and non-IR individuals (each dot represents an individual), using the cut-off value of the Matsuda index as reference (4.03) (*X*-axis) and its interaction with surrogate indices, anthropometrics measurements, glucose, and insulin levels (*Y*-axis). Matsuda index values below the cut-off of 4.03 are independently associated with IR. The right column shows the violin plots of the distribution of IR and non-IR individuals in relation to the different cut-off values and the diagnosis performance **(B, D, F, H, J, L, M, Ñ, P, R, T, V, X, Z)** (sensitivity and specificity) using the algorithmic approach for prediction of insulin resistance of the surrogate indices. The violin plots show the interquartile range distribution of IR and non-IR individuals. Homeostatic model assessment index (HOMA-IR), Quantitative insulin sensitivity check index (QUICKI), triglyceride-glucose (TyG) index, triglycerides-to-HDL-C ratio (TG/HDL-c), visceral adiposity index (VAI), TyG-waist circumference (TyG-WC), TyG-body mass index (TyG-BMI), lipid accumulation product (LAP), body mass index (BMI kg/m^2^), total fat mass (TFM %), android fat (AF %), waist circumference (WC), and waist-to-height ratio (WHtR).

Furthermore, this study determines the bivariate distribution of IR and non-IR individuals using the cutoff value of Matsuda index as reference obtained by the computational approach (independent variable), who represent the most accurately diagnostic performance surrogate index for predicting IR compared with the EHC technique, the gold standard method for the detection of IR (**Table 2**, **Figure 2**, and **Supplementary Figure 1**) (9).

**Table 2:** Diagnosis performance determined by computational approach using the Matsuda index as reference for predicting insulin resistance (IR) in young men of surrogate indices, lipid indices, anthropometric measurement, serum glucose, and insulin levels.

Index	Cutoff value*	Sensitivity	Specificity
**Matsuda**	4.03	1.00	1.00
**Body mass index (BMI) (kg/m^2^)**	28.69	1.00	1.00
**Waist circumference (WC) (cm)**	92.63	1.00	1.00
**WHtR**	0.53	1.00	1.00
**Total fat mass (TFM %)**	31.07	1.00	1.00
**Android fat (AF %)**	40.33	1.00	0.98
**HOMA-IR**	3.34	0.91	0.98
**Leptin (ng/mL)**	16.08	0.91	1.00
**LAR (ng/µg)**	1.17	0.84	1.00
**QUICKI**	0.33	0.98	0.96
**TyG**	4.60	0.73	0.77
**TG/HDL-c**	2.93	0.69	0.92
**TyG-WC**	427.77	1.00	1.00
**TyG-WHtR**	2.48	0.98	1.00
**TyG-BMI**	132.44	1.00	1.00
**LAP**	45.49	0.84	1.00
**VAI**	3.64	0.69	0.92
**Fasting glucose (mg/dL)**	85.92	0.6	0.65
**Glucose (mg/dL) 60′ OGTT**	98.02	0.69	0.81
**Glucose (mg/dL) 120′ OGTT**	82.10	0.51	0.77
**Fasting Insulin (µIU/mL)**	16.02	0.91	0.98

Diagnosis performance determined by computational approach was assessed using the Matsuda index as reference (cutoff value 4.03*). Homeostatic model assessment index (HOMA-IR), Quantitative insulin sensitivity check index (QUICKI), triglyceride-glucose (TyG) index, triglycerides-to-HDL-C ratio (TG/HDL-c), visceral adiposity index (VAI), TyG-waist circumference (TyG-WC), TyG-body mass index (TyG-BMI), TyG-waist-to-height ratio (TyG-WHtR), lipid accumulation product (LAP), leptin/adiponectin ratio (LAR), body mass index (BMI kg/m^2^), total fat mass (TFM %), android fat (AF %), waist circumference (WC), and waist-to-height ratio (WHtR).

Furthermore, the cutoff value used to discriminate IR, defined with the Matsuda index (4.03), and the diagnosis performance using the computational approach described above for the determination of IR in young men for surrogate indices, anthropometric measurement, leptin, serum glucose, and insulin levels are described in **Table 2** (dependent variable). Therefore, individuals with Matsuda index values below the cutoff of 4.03 were defined as insulin resistant (**Figure 2**).

On the other hand, the scatter plots in **Figure 2** and **Supplementary Figure 1** show the bivariate distribution segregated by the status of groups of IR and non-IR individuals using the cutoff value of Matsuda index as reference (midpoint of the separation window) (*X*-axis) in relation to the different cutoff values of IR/insulin sensitivity for surrogate IR indices, lipid indices, anthropometric measurement, leptin and serum insulin, and glucose level values (*Y*-axis) (left column of **Figure 2**). As it can be observed in **Figure 2**, low values of the Matsuda index are independently associated with the risks of IR (<4.03) (**Tables 1** and **2**, **Figure 2**, and **Supplementary Figure 1**). Additionally, the violin plots show the distribution of groups of IR and non-IR individuals in relation to the different cutoff values and the diagnosis performance (sensitivity and specificity) for the prediction of IR for surrogate indices, anthropometric measurement, leptin and serum glucose, and insulin levels (**Table 2**, **Figure 2**, and **Supplementary Figure 1**) (right column of **Figure 2**). Therefore, the computational approach determined the best diagnostic performance (sensitivity and specificity) and cutoff values to discriminate IR for BMI (1.00; 1.00; 28.69), WC (1.00; 1.00; 92.63), WHtR (1.00; 1.00; 0.53), TyG-WC (1.00; 1.00; 427.77), TyG-BMI (1.00; 1.00; 132.44), TyG-WHtR (0.98; 1.00; 2.48), TFM (%) (1.00; 1.00; 31.07), AF (%) (1.00; 0.98; 40.33), LAP (0.84; 1.00; 45.49), HOMA-IR (0.91; 0.98; 3.40), QUICKI (0.98; 0.96; 0.33), LAP (0.84; 1.00; 45.49), LAR (0.84; 1.00; 1.17), leptin (0.91; 1.00; 16.08), and fasting insulin (0.91; 0.98; 16.01) (**Table 2** and right column of **Figure 2**). Additionally, TyG (0.73; 0.77; 4.60), TG/HDL-c (0.69; 0.92; 2.93), VAI (0.69; 0.92; 3.64), fasting glucose (mg/dL) (0.60; 0.65; 85.92), glucose (mg/dL) 60′ OGTT (0.69; 0.81; 98.02), and glucose (mg/dL) 120′ OGTT (0.51; 0.77; 82.10) showed modest diagnostic accuracy derived from the computational approach (**Table 2**, right column of **Figure 2** and **Supplementary Figure 1**).

**Figure 3:**
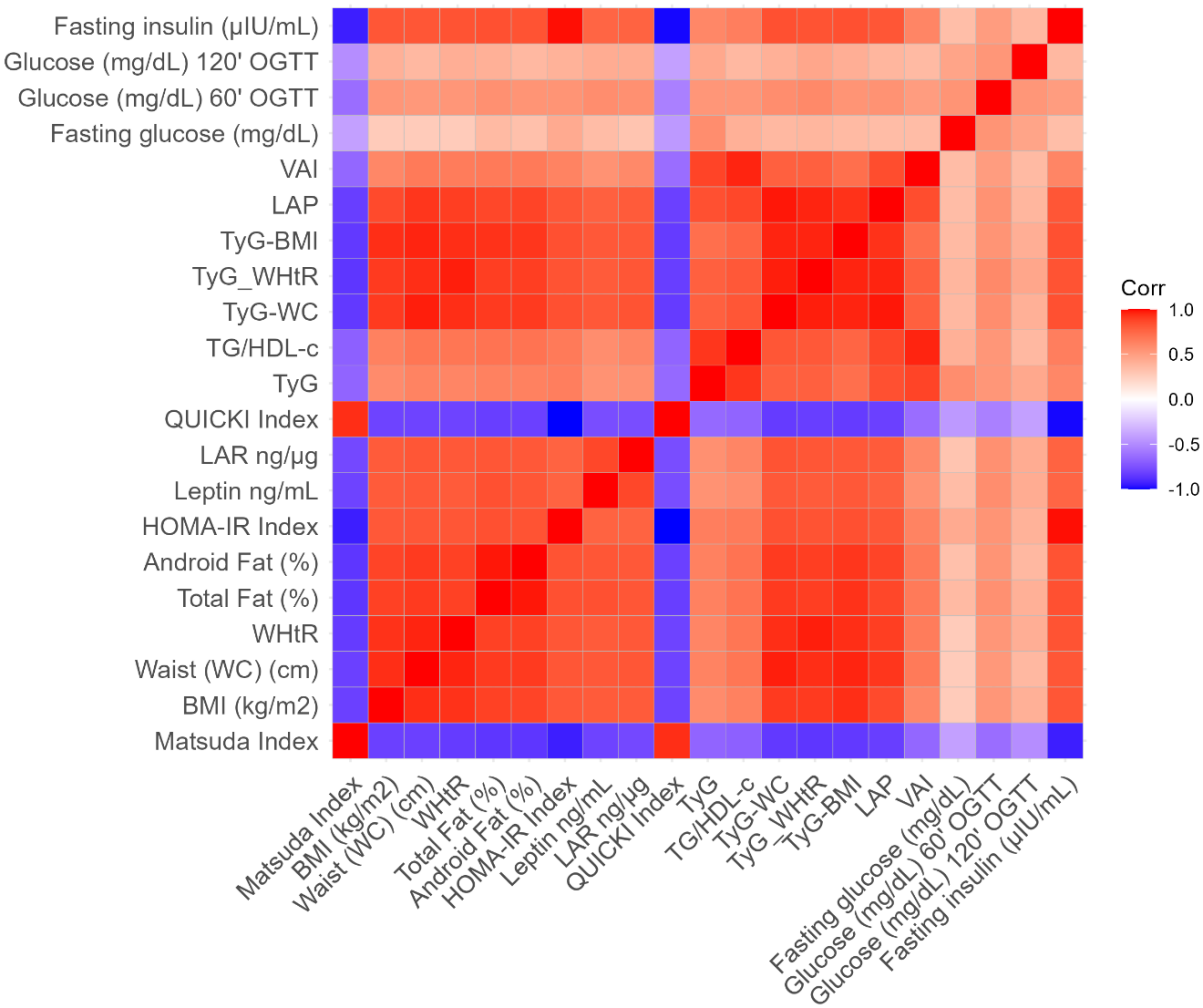
The Spearman correlation matrix of all pairs of variables included in the algorithm approach model was created and the correlation value was presented in the cells. The colors represent the magnitude of the Spearman correlation. Homeostatic model assessment index (HOMA-IR), Quantitative insulin sensitivity check index (QUICKI), triglyceride-glucose (TyG) index, triglycerides-to-HDL-C ratio (TG/HDL-c), visceral adiposity index (VAI), TyG-waist circumference (TyG-WC), TyG-body mass index (TyG-BMI), lipid accumulation product (LAP), body mass index (BMI kg/m^2^), total fat mass (TFM %), android fat (AF %), waist circumference (WC), and waist-to-height ratio (WHtR).

**Figure 4:**
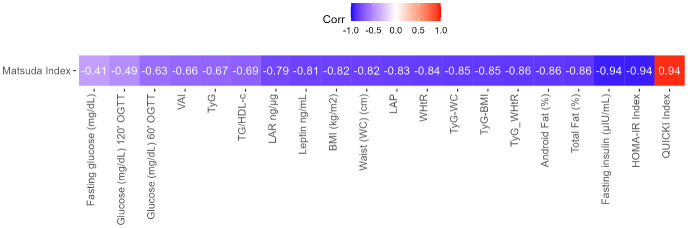
Vector of Matsuda’s correlations with different indices or variables. The vector was extracted from the correlation matrix and ordered by magnitude, since Matsuda was considered the gold standard among these indices. The colors represent the magnitude of the Spearman correlation. Homeostatic model assessment index (HOMA-IR), Quantitative insulin sensitivity check index (QUICKI), triglyceride-glucose (TyG) index, triglycerides-to-HDL-C ratio (TG/HDL-c), visceral adiposity index (VAI), TyG-waist circumference (TyG-WC), TyG-body mass index (TyG-BMI), lipid accumulation product (LAP), body mass index (BMI kg/m^2^), total fat mass (TFM %), android fat (AF %), waist circumference (WC), and waist-to-height ratio (WHtR).

The Spearman correlation matrix of all pairs of variables included in the model was created and the correlation value was presented in the cells (**Figure 3**). Additionally, the vector of Matsuda’s correlations with different indices or variables was extracted from the correlation matrix and ordered by magnitude, since Matsuda was considered the gold standard among these indices and colors represent the magnitude of the Spearman correlation (**Figure 4**).

Finally, given the exploratory character of the present study based on a mathematical model, there was no pre-specified inferential hypothesis. The nature of the study is multivariate for a binary classifier. Rajput et al. have recommended an effect size of 0.5 or higher to evaluate a decided sample size in machine learning applications; by having 20 correlations with Matsuda, a convenient threshold can be set that at least 95% of the correlations are greater than 0.5, and in our case, 19 of the 20 correlations were greater than 0.5 (45). The stability of the algorithm at such high correlations gave us the guarantee of its good performance for all pairs of variables involving Matsuda.

## Discussion

In the present study, using a computational approach, we determined the diagnosis accuracy and the cutoff values for the determination of IR with the Matsuda index as reference for surrogate indices of muscle and hepatic insulin sensitivity/resistance, lipid indices, anthropometric measures, hormonal levels, serum glucose, and insulin levels in non-diabetic young adult men. This approach can be used quickly, easily, and at a low cost in routine IR screening for preventive health services.

In this regard, BMI, TyG-WC, TyG-BMI, WHtR, WC, TyG-WHtR, TFM (%), AF (%), LAP, Leptin, LAR, HOMA-IR, QUICKI, and fasting insulin levels showed high diagnostic accuracy for the prediction of IR using the computational approach. Moreover, it is important to highlight that some cutoff values determined in this study using the computational approach are similar to those described in previous research, between TyG-WC, TyG-BMI, TyG-WHtR, WHtR, WC, BMI, HOMA-IR, leptin, LAR, and QUICKI indices and insulin levels, variables that are highly correlated with IR and T2DM (13, 30–32, 40, 46–52). In contrast, consistent with previous studies, TyG, TG/HDL-c, VAI, fasting glucose (mg/dL), glucose (mg/dL) 60′ OGTT, and glucose (mg/dL) 120′ OGTT displayed moderate diagnostic accuracy for detecting IR, as described elsewhere (14, 29–33, 47, 51, 53). It must be noted that the cutoff value for the TyG index obtained in the present study (4.60) has the same value as described for the first time by the authors who proposed this index (29, 30). Moreover, the cutoff value for the TG/HDL-c index (2.93) obtained in the present study resembles the value described by Wakabayashi et al. (33). It is worth noting that different studies have demonstrated that TyG and TG/HDL-c indices are significantly associated with the risk of T2DM, stroke, and cardiovascular mortality (54, 55).

Researchers have found that visceral obesity accompanied by hypertrophy and hyperplasia of adipose tissue is characterized by low-grade chronic inflammation, IR, and different metabolic alterations (56, 57). Different studies have shown marked differences and guidelines for unification criteria in relation to the cutoff values and diagnosis performance for surrogate indices and variables that allow to predict IR, particularly those related to anthropometric measurement, and hormonal and biochemical parameters according to age, race, and ethnicity (20, 52, 58–61). Thus, although WC is gender and race/ethnicity specific, its use as a surrogate index for the determination of IR in population studies is limited because it may lead to the underestimation or overestimation of IR prediction; therefore, this index should be adjusted to height for a robust and universal use as a surrogate index for predicting IR (20, 40, 62). Similarly, it is important to highlight that BMI is age, gender, and race/ethnicity specific to predict IR, and countless cutoff values have been described in different population studies; therefore, this index should be carefully applied in personalized medicine rather than in population studies for predicting IR (44, 63, 64). Additionally, Teresa Vanessa Fiorentino et al. have demonstrated that the LAP index showed higher diagnostic accuracy compared with TyG, TG/HDL-C ratio, and VAI indices in detecting IR and CVDs (65). In addition, Nayeon Ahn et al. have demonstrated that VAI, LAP, and TyG showed high discrimination performance in the diagnosis of individuals with prediabetes and T2DM (32, 66).

In contrast, in the present study, the WHtR cutoff value determined via a computational approach is highly consistent with data from previous research findings according to age and race/ethnicity (40, 67–71). Moreover, it has been demonstrated in different studies that the surrogate indices TyG-WC, TyG-WHtR, and TyG-BMI presented high diagnostic accuracy for predicting IR, presented highly conserved cutoff values across different human populations studies, and can be easily calculated from routine laboratory tests, as described elsewhere (12, 29, 30). Furthermore, it is important to highlight that different studies have shown that TyG-WC, TyG-WHtR, and TyG-BMI indices are strong predictors of IR, T2DM, and metabolic diseases such as hepatic steatosis (72, 73).

On the other hand, different studies have shown a stronger relationship between TFM (%) and AF (%) with IR (74, 75). Furthermore, abdominal-android and visceral fat accumulation is strongly associated with the risk of CVD, T2DM, stroke, and several negative health outcomes (76, 77). Therefore, in this study, using the computational approach, and having the Matsuda index as reference, the cutoff values for IR prediction were determined for TFM (%) and AF (%). However, high variation between body fat distribution and IR has been demonstrated across gender and ethnic/racial population studies (75, 76). Additionally, different studies have demonstrated that obese individuals often present with hyperleptinemia, chronic low-grade systemic inflammation, and IR (78). It has been reported that LAR is associated with IR and metabolic syndrome (49). In this regard, in the present study, the LAR index was determined by computational approximation using the Matsuda index as reference, with similar findings to those described elsewhere (79).

Moreover, in the present study, the diagnosis accuracy performance and the cutoff values for the prediction of IR for HOMA-IR and QUICKI indices were determined via a computational approach. The results showed a high-accuracy performance to determine IR for both indices; however, the HOMA-IR index presents multiple cutoff values across racial/ethnic groups of population studies (80, 81). In contrast, the QUICKI index cutoff value is highly conserved in different gender and racial/ethnic population studies (0.33) despite fasting glucose and insulin levels being common variables used to determine both indices (52, 82).

Previous reports have demonstrated that the QUICKI index is one of the simplest and best evaluated and validated surrogate indices with higher predictive power and accuracy for determining insulin sensitivity/resistance and the development of diabetes (24, 83). In addition to the QUICKI index, TyG-WC, TyG-WHtR, and TyG-BMI indices present high predictive accuracy and are cost-effective to use quickly and easily for the early detection of IR screening, monitoring, and evaluating therapeutic interventions and preventive medicine in the general population.

It is important to highlight that worldwide population migrations have been increasing significantly for different reasons, such as political, demographic, economic, and social causes, and usually happen within a country, across borders, and across continents (84). Migration studies have demonstrated that the highest international migration rates occur in Oceania (22%), North America (16%), and Europe (12%); low migration rates occur in Asia (1.8%), Africa (1.9%), and Latin America and the Caribbean (2.3%) (84). In this way, migration from low- and middle-income countries to high-income countries exposes migrant populations to epigenetic modification that might lead to the development of deleterious effects on the health of individuals mostly through NCDs such as obesity, diabetes, hypertension, stroke, infectious disease, cancers, and mental disorders (85). Furthermore, it has been generally demonstrated that the most commonly used IR indices might vary with age, gender, and ethnicity, and thus, healthcare services have to take into consideration that these indices must be implemented for precise patient monitoring to detect and diagnose medical conditions in real time (59, 86). Thus, the analysis of cutoff values and diagnosis performance for different surrogate indices through a computational approach and using the Matsuda index as reference could contribute in the future to implement health policies and preventive care strategies for the rapid, massive, and low-cost identification of patients with IR in order to reduce the high costs of chronic and NCDs.

The main strength of our study is having used for the first time the Matsuda index as reference for detecting the cutoff values and diagnosis performance to determine the risk of IR of different surrogates’ indices using an accurate, robust, and flexible computational approach. In addition, it is worth highlighting that the Matsuda index is a whole-body insulin sensitivity surrogate index with high diagnostic performance, when compared with the gold standard method for assessing insulin sensitivity in humans, the EHC technique. Additionally, in the present study, the cutoff values determined for the different surrogate indices, which showed the highest diagnosis performance using a computational approach, are in agreement with human population studies designed with a large number of individuals and taking into consideration the ethnicity/race, age, and gender, as described above. Furthermore, the validity of the current methodology and results is strongly supported by anthropometric parameters such as total fat, visceral fat, WC and BMI, serum insulin, leptin, and adiponectin levels, and by clinical variables that have been used previously to determine IR and are in accordance with the different cutoff points established for the different indices in the current study.

On the other hand, this study has some limitations, including the exploratory cross-sectional design with case and control selection of the individuals (obese and healthy men), and the development of the algorithm only in adult volunteer men selected. Other studies should be developed in the future taking into account demographic variables such as age, gender, ethnicity, and all the spectrum of IR. With a larger sample size and consecutive recruitments, we will expect to overcome those limitations in order to know the real diagnosis performance of surrogate indices of IR. Finally, the present study aims to contribute to the prevention of NCDs such as IR/insulin sensitivity in any context, quickly and at a low cost, taking into account that through the algorithmic approach, the cutoff points and diagnostic performance must be established for the different indices of IR and according to ethnicity/race, age, and gender.

## Conclusions

In this study, a computational approach was used to determine the diagnosis accuracy and the cutoff values for different surrogate indices to determine IR using the Matsuda index as reference. Some of these indices are easy to implement in daily clinical practice, showing high diagnostic accuracy, with similar cutoff values for the prediction of IR to those indices described in previous research. Therefore, TyG-WC, TyG-BMI, WHtR, TyG-WHtR, and QUICKI must be studied and adjusted for age, gender, and race/ethnicity for estimating insulin sensitivity/resistance using a computational approach.

## Data availability statement

Data generated during the study was included in this article. Also, the data of the current study are available from the corresponding author upon request.

## Ethics statement

This protocol was approved by the Ethics Committee of the School of Medicine – Universidad Nacional de Colombia (protocols B.FM.1.002- CE-0194-22 and B.FM.1.002- CE-081-22). The studies were conducted in accordance with the local legislation and institutional requirements. The participants provided their written informed consent to participate in this study.

## Author contributions

VM-S: Conceptualization, Writing – review & editing. AL-F: Conceptualization, Formal analysis, Writing – review & editing. CE-P: Conceptualization, Formal analysis, Writing – review & editing. ÁB-C: Data curation, Writing – review & editing. MG: Methodology, Writing – review & editing. GO-R: Conceptualization, Writing – review & editing. RF-V: Writing – original draft. JP-F: Conceptualization, Formal analysis, Writing – review & editing. LM-A: Conceptualization, Formal analysis, Writing – review & editing. JR-R: Data curation, Writing – review & editing. MM-P: Conceptualization, Formal analysis, Writing – review & editing. SC-C: Writing – original draft. EL: Data curation, Formal analysis, Validation, Writing – original draft. CR-M: Data curation, Formal analysis, Validation, Writing – review & editing. AD-C: Data curation, Formal analysis, Methodology, Writing – review & editing. AR-P: Writing – original draft. JC: Writing – original draft.
