# Supplementary material for: Surrogate indices of insulin resistance using the Matsuda index as reference in adult men—a computational approach
Source: Front Endocrinol (Lausanne). 2024 Apr 23;15:1343641. doi: 10.3389/fendo.2024.1343641 (PMC11075368; doi:10.3389/fendo.2024.1343641)
Supplement: Supplementary file 1 [file Presentation_1.pdf]

Supplementary Material

Surrogate Indices of Insulin Resistance using as Reference the Matsuda Index in Adult Men - A Computational Approach

*Víctor Antonio Malagón – Soriano[1], *Andres Julian Ledezma – Forero[1], Cristian Felipe Espinel – Pachon[1], Álvaro Javier Burgos – Cárdenas[1], Maria Fernanda Garcés[2], Gustavo Eduardo Ortega - Ramírez[3], Roberto Franco – Vega[3], Jhon Jairo Peralta – Franco[3], Luis Miguel Maldonado – Acosta[3], Jorge Andres Rubio – Romero[4], Manuel Esteban Mercado – Pedroza[4], Sofia Alexandra Caminos – Cepeda[5], Ezequiel Lacunza[6], Carlos Armando Rivera - Moreno[2], Aquiles Enrique Darghan - Contreras[2], Ariel Iván Ruiz – Parra[4], Jorge Eduardo Caminos[2]

[1]Department of Internal Medicine, [2]Department of Physiology, [3]Endocrinology Unit - Department of Internal Medicine and [4]Department of Obstetrics & Gynecology, School of Medicine Universidad Nacional de Colombia, Bogotá 11001, Colombia. [5]School of Medicine, Universidad Pompeu Fabra; Barcelona 08002, Spain. [6]Centro de Investigaciones Inmunológicas Básicas y Aplicadas (CINIBA), Facultad de Ciencias Médicas, Universidad Nacional de La Plata, La Plata 1900, Argentina.

*V.A.M.S and A.J.L.F contributed equally to this work.

Correspondence: Jorge Eduardo Caminos, MSc. PhD., Department of Physiology, School of Medicine, Universidad Nacional de Colombia, Carrera 30 No. 45-03, Edificio 471 Oficina 406, Bogotá, Colombia, E-mail: jecaminosp@unal.edu.co

Short running head: Surrogate Indices to determine Insulin Resistance in Young Men – A Computational Approach

1. Supplementary Figure 1:

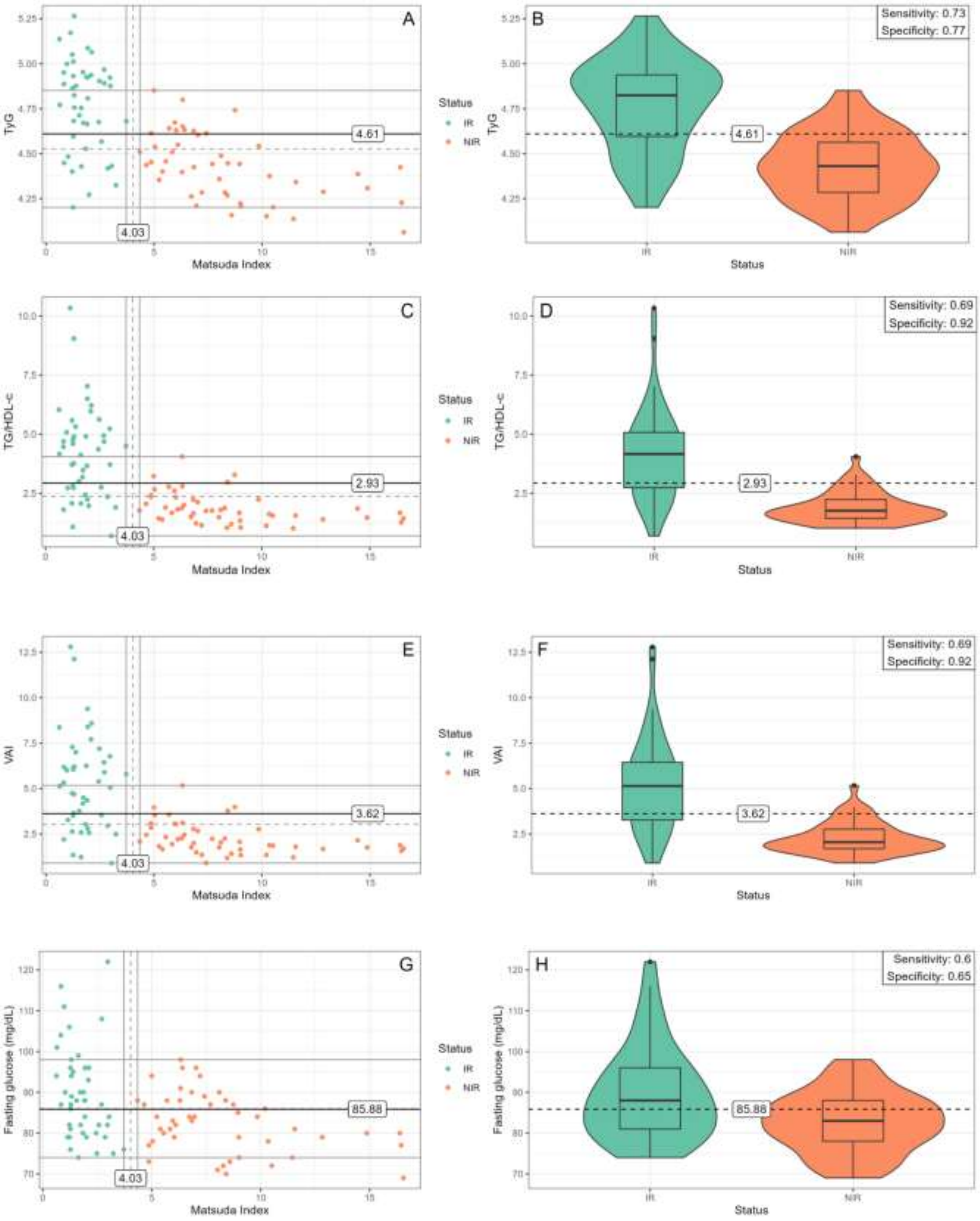

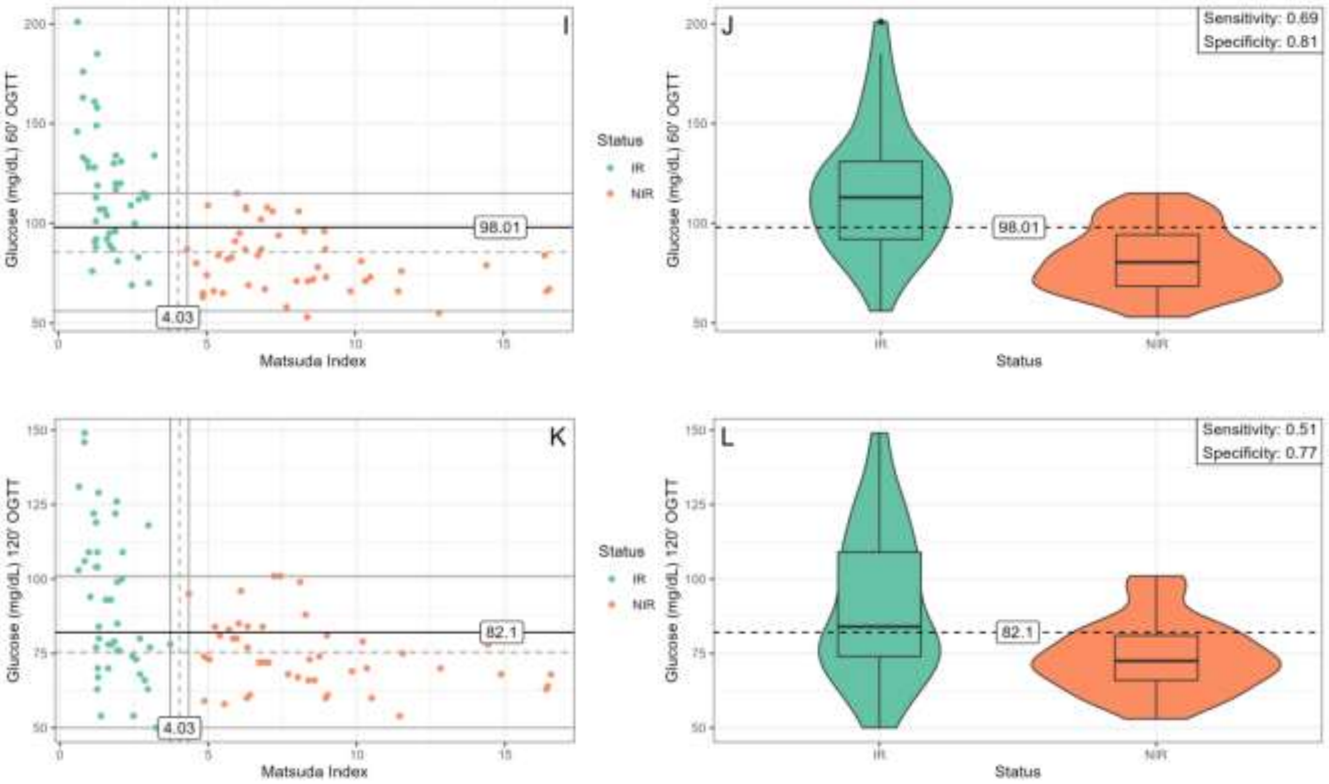

Triglyceride-glucose (TyG) index, triglycerides-to-HDL-C ratio (TG/HDL-c), visceral adiposity index (VAI)

Supplementary Figure 1: The figures in the left column show the scatter plot depicting the bivariate distribution segregated by status of IR and non – IR individuals (each dot represents an individual), using the cut-off value of Matsuda index as reference (4.03) (X-axis) and its interaction with surrogate indexes, anthropometrics measurements, glucose and insulin levels (Y-axis). Matsuda index values below the cut-off of 4.03 are independently associated with IR. The right column shows the violin plots of the distribution of IR and non – IR individuals in relation to the different cut-off values and the diagnosis performance (sensitivity and specificity) using the algorithmic approach for prediction of insulin resistance of the surrogate indexes.

2. Computational Algorithm Approach for determination of diagnosis performance and cut-off values for surrogate indices.

In the GitHub repository address appears the algorithm developed for the process of optimization of sensitivity and specificity of each variable that was contrasted with the Matsuda index. A double standardization was made to the data, one to give a spatial context to each observation for the construction of weights matrices to delay the coordinates of the two-dimensional space that formed the pair of variables, and the second, to adjust variability of each of the variables. The generated scattering diagram evidenced a monotonous behavior of each pair of variables rather than linear thus we used as weighted lags, the quadratic Spearman correlation coefficient. Finally, the iterative process was done to adjust for observations that could affect the stability of the optimum by a procedure of eliminating one row at a time. For each port of call facing the Matsuda index, the values of sensitivity, specificity and cut-off values in the separation window or overlap of the variable contrasted with Matsuda were recorded.

The following link shows the Computational Algorithm Approach for determination of diagnosis performance and cut-off values for surrogate indices:

https://github.com/CarlosRivera1212/Cut_off_Matsuda-

3. Description of insulin surrogate indices formulas

Matsuda Index = $10.000/\sqrt{\text{Go X Io X mean G X mean Insulin}}$

HOMA index = $(G_o \times I_o)/22.5$

QUICKI = $1/(\log G_o + \log I_o)$

BMI = kg/m^2

TG/HDL-C = Triglycerides (mg/dL) / HDLc (mg/dL)

TyG index = Ln [fasting glucose (mg/dL) × triglycerides (mg/dL)] /2

TyG - BMI = [Ln [fasting glucose (mg/dL) ×triglycerides (mg/dL)] /2] × BMI

TyG - WC= [Ln [fasting glucose (mg/dL) ×triglycerides (mg/dL)] /2] × WC (cm).

WHtR = Waist (cm) / Height (m)

TyG-WHtR = TyG / WHtR

LAP= [WC (cm) − 65) × (triglycerides (mmol/L)] for men

VAI= (WC (cm)/ (39.68+ (1.88×BMI))) × (triglycerides (mmol/L)/1.03) × (1.31/HDL-C (mmol/L)) for men

Abbreviations

I_0 - Fasting insulin

G_0 -Fasting glucose

G mean - Mean serum glucose

I mean - Mean serum insulin

TyG - Triglycerides and glucose index

TG/HDL-C - Triglycerides-to-HDL-c ratio

WC cms - Waist circumference

TyG-BMI - TyG-body mass index

TyG-WC - TyG-waist circumference

WHtR - Waist-to-height ratio

QUICKI - Quantitative insulin sensitivity check index

TyG-WHtR - TyG-waist-to-height ratio

LAP - Lipid accumulation product

VAI - Visceral adiposity index
